# Prehospital triage accuracy in a criteria based dispatch centre

**DOI:** 10.1186/s12873-015-0058-x

**Published:** 2015-10-27

**Authors:** Fabrice Dami, Christel Golay, Mathieu Pasquier, Vincent Fuchs, Pierre-Nicolas Carron, Olivier Hugli

**Affiliations:** Dispatch centre, State of Vaud (Fondation Urgences-Santé), César-Roux 31, 1005 Lausanne, Switzerland; Department of Emergency Medicine, University Hospital Center (CHUV), Bugnon 46, 1011 Lausanne, Switzerland; Faculty of Biology and Medicine, University of Lausanne, Lausanne, Switzerland

**Keywords:** EMS, Criteria-based dispatch, Accuracy, Benchmarking

## Abstract

**Background:**

Priority dispatch accuracy is a key issue in optimizing the match between patients’ medical needs and pre-hospital resources. This study measures the accuracy of a Criteria Based Dispatch (CBD) system, by evaluating discrepancies between dispatch priorities and ambulance crews’ severity evaluations.

**Methods:**

This is a retrospective study conducted from January 2011 to December 2011. We ruled that a National Advisory Committee for Aeronautics (NACA) score > 3 (injuries/diseases which can possibly lead to deterioration of vital signs) to 7 (lethal injuries/ diseases) should require a priority dispatch with lights and siren (L&S), while NACA scores < 4 should require a priority dispatch without L&S. Over triage was defined as the proportion of L&S dispatches with a NACA score < 4, and under triage as the proportion of dispatches without L&S with a NACA score > 3.

**Results:**

There were 29,008 primary missions in 2011, 1122 were excluded. Of the 15,749 L&S missions, 12,333 patients had a NACA score < 4, leading to an over triage rate of 78 %; 561 missions out of 12,137 missions without L&S had a NACA score > 3, leading to an under triage rate of 4.6 %. Sensitivity was 86 % (95 % confidence interval: 85.6–86.4 %), specificity 48 % (47.4–48.6 %), positive predictive value 21.7 % (21.2–22.2 %), and negative predictive value 95.4 % (95.2–95.6 %).

**Conclusion:**

The rates of over triage and under triage in our CBD are 78 and 4.6 % respectively. The lack of consistent or universal metrics is perhaps the most important limitation in dispatch accuracy research. This is mainly due to the large heterogeneity of dispatch systems and prehospital emergency system.

## Background

Priority dispatch accuracy is a key issue in optimizing the match between patients’ medical needs and pre-hospital resources [1]. Although it is less studied than early cardiac arrest identification and telephone-CPR instructions, this topic is a major issue for dispatch centres as they all try to achieve the most efficient use of their resources. Over triage from dispatch centres represents an immediate response with lights and sirens (L&S) for a low-acuity case. It consumes limited resources, may increase costs and causes a shortage of ambulances for high-acuity emergencies; it could also endanger emergency medical services (EMS) workers and the general population, with ambulances running hot [2] with no or little benefit to the patient [3]. On the other hand, under triage from dispatch centres represents an inappropriately low response without priority signs in the presence of an acute case. Although this has not been documented at the dispatch level, it may place the patients at risk of transient unmet medical needs and delayed access to the appropriate level of care as it is for trauma patients from field triage [4].

Today there is no consensus on the accepted percentage of over and under triage at the dispatch level because of a very high heterogeneity in EMS. Different types of dispatch systems are used within the world: medical priority dispatch system (MPDS), which is the most widespread, is mainly found in North America [5, 6] and in the UK [7]; Criteria Based Dispatch (CBD) systems are almost exclusively located in European countries (including Denmark [8], Norway [9], Belgium [10], and regions of Switzerland [11]); physician dispatch is used in France [12]. There are also different types of resources available within the world of Emergency Medical Services (EMS): presence of advanced life support teams and/or basic life support teams, with or without first-responders or pre-hospital emergency physicians, which may influence dispatch protocols.

Our hypothesis is our dispatch has a high level of overtriage. The aim of this study was to evaluate the accuracy of a CBD system, by evaluating discrepancies between the dispatch priorities and ambulance crews’ severity evaluations, and to quantify the over and under triage.

## Methods

### Setting

This study was conducted throughout the State of Vaud in the French-speaking region of Switzerland, where a centralized pre-hospital medical dispatch centre serves a population of 750,000 and handles over 80,000 calls per year. The dispatch centre is staffed by registered nurses and certified paramedics with at least 5 years of field experience. It is a CBD system based on caller’s description of symptoms. All calls answered are assigned a keyword from a pre-determined list. Not only must dispatchers categorize every call, it is also mandatory for them to inquire on the victim’s ‘state of consciousness’ and ‘quality of breathing’ to detect cardiac arrest. Dispatchers rely on their own medical background and personal experience to ask the questions they deem appropriate to perform the interview. Each call is processed by the same dispatcher from the beginning (interview) to the end (dispatch). When appropriate, they deliver telephone-guided life-saving maneuvers to bystanders [13]. They benefit from 40 h of continuing education every year and are regularly evaluated to ensure that our quality standards are met. In Switzerland, priority 1 (immediate departure with L&S) is required only if dispatchers believe that there is a vital risk for the patient. Priority 2 is an immediate departure without L&S and priority 3 is a delayed departure [14]. Ambulances assigned a priority level 1 or 2 are staffed with at least one paramedic, while ambulances assigned a priority level 3 may be staffed with emergency medical technicians only.

Our pre-hospital network is a three-tier system. Ambulance crews dispose of state protocols for autonomous intravenous access, cardiopulmonary resuscitation procedures, defibrillation, and emergency medication administration [15]. Pre-hospital emergency physicians may be dispatched to the site by the call centre or later at the request from paramedics, either by ground or by helicopter.

When dealing with a low-acuity case that may not need a transport, our dispatchers can transfer the caller to the state’s nurse-counselling dispatch which provides medical advice or can dispatch an on-call general practician 24/7 within an hour. Those cases are not included in this study, unless the on-call physician decided a transport was necessary after visiting the patient.

Most of the scheduled and non-urgent transports from nursing homes to the hospitals or between hospitals do not undergo an evaluation by our dispatch centre as they are directly organized by the nursing home or hospital with the ambulance company. Those cases are not included in this study.

### Study design

We retrospectively studied the registry database of our dispatch centre, from January 1^st^ 2011 to December 31^st^ 2011. The registry records all dispatches in our State; pre-hospital teams input their data at the end of each mission. We excluded secondary missions (inter-hospital transfers), helicopter missions without ambulance already on site, missions aborted, and those with missing data.

**Fig. 1:**
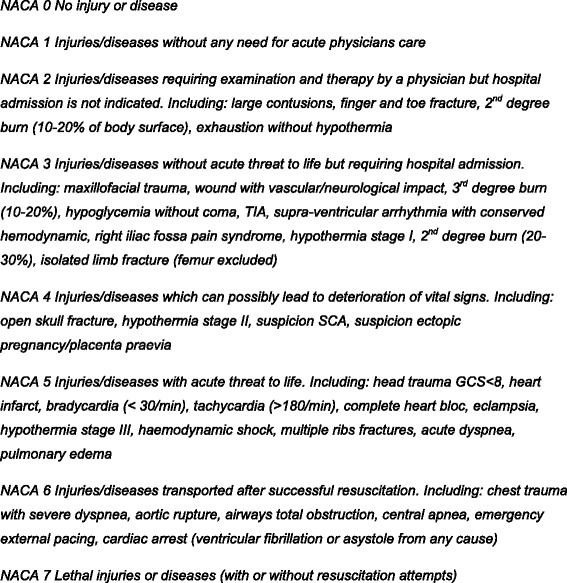
National Advisory Committee for Aeronautics (NACA) score revised by the State of Vaud, Switzerland (2005)

The data collected from each mission were the priority chosen by dispatcher, the keyword or determinant chosen to qualify the situation, and the severity of the condition assessed by the pre-hospital crews according to the National Advisory Committee for Aeronautics (NACA) score and transmitted to the dispatch at the end of the mission [16, 17]. The NACA score is an eight-level scale to assess pre-hospital severity status; the score is defined by the most serious clinical state experienced at any given time during the mission. (Fig. 1) The NACA score is commonly used in western Europe in pre-hospital emergency medicine and is significantly correlated with survival [18, 19]. The NACA score enables categorization of the victims’ condition, allows for statistical reviews of the type of injuries and illnesses treated, and illustrates the case-mix of pre-hospital health-care professionals. This scoring is mandatory in Switzerland for all ambulance and helicopter missions.

We initially ruled that a NACA score of 4 (injuries/diseases which can possibly lead to deterioration of vital signs) to 7 (lethal injuries or diseases, with or without resuscitation attempts) should require a P1 dispatch priority (with L&S), while NACA scores under 4 should require a P2 or P3 dispatch priority (without L&S). Over triage was defined as the proportion of P1 dispatches for patients with NACA score <4, and under triage as the proportion of P2 and P3 dispatches for patients with NACA score >3. The main outcome of the study was to measure the correlation between priority dispatch assigned by the dispatcher (P1–P2–P3) and the NACA score given by EMS personnel. The score given by the EMS personnel was considered the gold standard and therefore defined over and under triage by the dispatcher.

### Statistics

Simple descriptive statistics were used. Sensitivity, specificity, positive and negative predictive values, averages, and percentages were calculated with Microsoft Office Excel 2007.

## Results

**Fig. 2:**
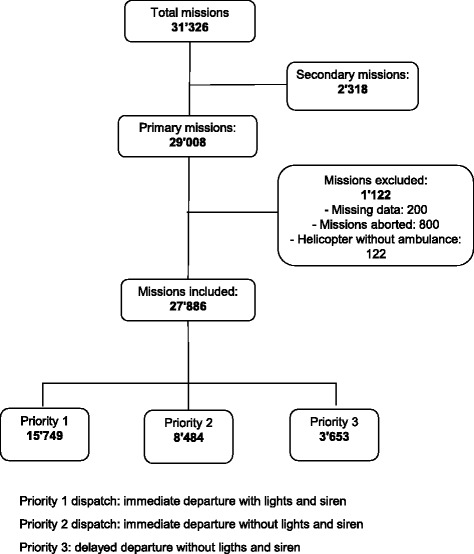
Flow chart

There were 29,008 primary missions in 2011 of which 1122 were excluded, leaving 27,886 missions included in our study: 15,749 P1 (57 %), 8484 P2 (30 %), 3653 P3 (13 %) (Fig. 2).

Of the 15,749 P1 missions 12,333 patients had a NACA score <4, leading to a rate of over triage of 78 %; 561 missions out of 12,137 P2/P3 missions had a NACA score >3, leading to an under triage of 4.6 %. Sensitivity of our triage was 86 % (95 % confidence interval: 85.6–86.4 %), specificity 48 % (47.4–48.6 %) while positive predictive value (PPV) was 21.7 % (21.2–22.2 %) and negative predictive value (NPV) 95.4 % (95.2–95.6 %) (Table 1).

**Table 1:** Priority dispatch and NACA score

Missions	Total [*n* (%)]	P1 [*n* (%)]	P2 [*n* (%)]	P3 [*n* (%)]	Correct correlation [*n* (%)]
NACA 0	332 (1.2)	184 (55.4)	128 (38.6)	20 (6.0)	148 (44.6)
NACA 1	1’247 (4.5)	830 (66.5)	371 (29.8)	46 (3.7)	417 (33.4)
NACA 2	7’122 (25.6)	4’285 (60.2)	2’228 (31.3)	609 (8.5)	2’837 (39.8)
NACA 3	15’208 (54.5)	7’034 (46.2)	5’290 (34.8)	2’884 (19.0)	8’174 (53.7)
NACA 4	2’480 (8.9)	2’034 (82.0)	376 (15.2)	70 (2.8)	2’034 (82.0)
NACA 5	867 (3.1)	767 (88.5)	79 (9.1)	21 (2.4)	767 (88.5)
NACA 6	203 (0.7)	196 (96.5)	5 (2.5)	2 (1.0)	196 (96.6)
NACA 7	427 (1.5)	419 (98.1)	7 (1.7)	1 (0.2)	419 (98.1)
Total	27’886 (100)	15’749 (56.5)	8’484 (30.4)	3’653 (13.1)	14’992 (53.8)

OVER TRIAGE = P1 dispatch with NACA <4 (false positive) / all P1 dispatch (false positive + true positive) = 12’333/15’749 = 78 %

UNDER TRIAGE = P2 or P3 dispatch with NACA >3 (false negative) / all P2 or P3 dispatch (false negative + true negative) = 561/12’137 = 4.6 %

Sensitivity was calculated as true positives/(true positives + false negatives); specificity, as true negatives/(false positives + true negatives)

Positive predictive value (PPV) was calculated as true positives/(true positives + false positives); negative predictive value (NPV), as true negatives/(true negatives + false negatives)

Sensibility = TP / (TP + FN) = 86 % = 95 % CI (85.6–86.4)

Specificity = FN / (FN + FP) = 48 % = 95 % CI (47.4–48.6)

PPV = TP/(TP + FP) = 21.7 % = 95%CI (21.2–22.2)

NPV = TN/(TN + FN) = 95.4 % = 95 % CI (95.2–95.6)

The most frequent scores attributed at the end of the missions were NACA 2 and 3 (54.5 % and 25.6 %, respectively). There was a majority of P1 dispatch in all NACA categories (mean: 56.5 %), ranging from 46.2 % for NACA 3 to 98.1 % for NACA 7. According to our definition of concordance, dispatcher and EMS agreed on priority in 53.8 % of missions (P1 for NACA score >3 and P2/P3 for NACA score <4). High-acuity cases (NACA >3) represented 14 % of all missions (Table 1). Paediatric cases (<18 years old) represented only 5 % of our case mix. Table 2 resumes the use of dispatch keywords in 2011. “Undefined problem” represents 60 % of our case-mix.

**Table 2:** Keywords used in 2011

Keyword	Missions [*n* (%)]
Undefined problem	16’739 (60.1)
Public place	3255 (11.7)
Dyspnoea	1’807 (6.4)
Disturbance of consciousness	1’768 (6.3)
Chest pain	1’449 (5.2)
Unconscious	1’364 (4.9)
Acute stroke < 5 h	428 (1.5)
Child (accident)	373 (1.3)
Intoxication	169 (0.6)
Child (dyspnoea)	98 (0.4)
Hypotension (symptomatic)	70 (0.3)
Fall above 3 m	60 (0.2)
Penetrating injury	46 (0.2)
Delivery (imminent)	45 (0.2)
Incarcerated	42 (0.2)
Prevention (fire)	35 (0.1)
Fall above 5 m	34 (0.1)
Impossible access for ambulance (mountains)	25 (0.1)
Demand from on-call physician	15 (0.1)
Burns (limited)	14 (0.1)
Child (unconscious)	9 (0.0)
Limb’s amputation	8 (0.0)
Burns (extensive, >10 %)	6 (0.0)
Drowning	6 (0.0)
Ejected	5 (0.0)
Para/tetraplegia	4 (0.0)
Car accident (>3 patients)	3 (0.0)
Explosion	2 (0.0)
Electrocution	2 (0.0)
Anaphylactic reaction	2 (0.0)
Diving accident	2 (0.0)
Accident in a tunnel	1 (0.0)
TOTAL	27’886 (100)

Most of the over triage concerns NACA 3 (57 %) and keywords ‘undefined problem’ (4676; 38 %), ‘public place’ (2847; 23 %), ‘disturbance of consciousness’ (1353; 11 %), ‘chest pain’ (947; 8 %), ‘dyspnoea’ (894; 7 %), ‘unconscious’ (676; 6 %) (Table 3).

**Table 3:** Over triage by keywords

	Total [*n* (%)]	NACA 0 [*n* (%)]	NACA 1 [*n* (%)]	NACA 2 [*n* (%)]	NACA 3 [*n* (%)]
N (P1 over triage)	12’333 (100)	184 (1.5)	830 (6.7)	4’285 (34.8)	7’034 (57.0)
Undefined problem	4’676 (37.9)	71	325	1’640	2’640
Public place	2’847 (23.1)	53	226	1’289	1’279
Disturbance of consciousness	1’353 (11.0)	24	93	407	829
Chest pain	947 (7.7)	4	26	220	697
Dyspnoea	894 (7.3)	11	67	243	573
Unconscious	676 (5.4)	14	45	176	441
Demand from on-call physician	247 (2.0)	1	5	45	196
Others	693 (5.6)	6	43	265	379

Most of the under triage concerns NACA 4 (79.5 %) with keywords ‘undefined problem’ (469; 83.6 %), ‘dyspnoea’ (54; 9.6 %) and ‘disturbance of consciousness’ (21; 3.7 %) (Table 4).

**Table 4:** Under triage by keywords

	Total [n (%)]	NACA 4 [n(%)]	NACA 5 [n(%)]	NACA 6 [n(%)]	NACA 7 [n(%)]
N (P2 + P3 under triage)	561 (100)	446 (79.5)	100 (17.8)	7 (1.3)	8 (1.4)
Undefined problem	469 (83.6)	377	80	7	5
Dyspnoea	54 (9.6)	40	14	0	0
Disturbance of consciousness	21 (3.7)	14	6	0	1
Chest pain	7 (1.2)	7	0	0	0
Public place	4 (0.7)	4	0	0	0
Acute stroke	2 (0.4)	2	0	0	0
Intoxication	2 (0.4)	2	0	0	0
Prevention	2 (0.4)	0	0	0	2

## Discussion

Our rate of over triage is 78 % and our rate of under triage is 4.6 %. There are published recommendation rates of over and under triage in pre-hospital trauma field medicine by the American College of Surgeons Committee on Trauma (5–10 % of under triage and 30–50 % of over triage) [20], but we are still missing such consensus on acceptable rates and objectives in medical dispatch. Thirty-one experts met in 2004 to establish standards on EMS studies, but specifically no agreement was found on over and under triage in particular [21].

Some looked for concordance between dispatch priorities and emergency department (ED) evaluation [22], or between dispatch, EMS and ED [23]. We decided to compare dispatch priorities and EMS field findings only, as EDs’ evaluation cannot take into account any significant change in the patient’s condition due to time and pre-hospital intervention. Priority dispatch should be evaluated by the first professional on site, so the impact of elapsed time from dispatch to clinical evaluation is minimal. Treatments provided by EMS may contribute to the improvement of the patient’s clinical condition, which will also interfere with the evaluation by ED personal of dispatch priority.

Benchmarking remains difficult even when looking only at studies that did compare dispatch priorities and EMS findings rather than ED evaluations. Dispatch centres (CBD, MPDS, physician dispatch) and EMS systems (two or three tiers) are very heterogeneous. Although all previous studies have dealt with the same metric issue (high versus low-acuity cases) there is no consensus on their definition. Therefore criteria to define a concordance between dispatch priority and the clinical findings on the field by EMS (gold standard) differ widely.

Table 5 presents results from previous studies dispatch over and under triage. Those examples show that not only are dispatch criteria different between studies, but also case acuity measurement tools by on field EMS. Therefore it is not possible to conclude that one system may be more efficient than another, whatever rates of over and under triage are published. Nevertheless, it is of prime importance for dispatch centres to publish their results as this may allow benchmarking and, with time, permit the reaching of an international consensus on dispatch accuracy.

**Table 5:** Previous results on dispatch over and under triage

	Over triage	Under triage	Sensitivity	Specificity	PPV	NPV
Criteria Based Dispatch						
Korram-Manesh A, et al. [23]	73 %	3.5 %	NA	NA	NA	NA
Ek B, et al. [33]	NA	NA	95.9 %	15.4 %	88.5 %	29.1 %
Medical Priority Dispatch						
Lu TC, et al. [34]	62.9 %	8.1 %	NA	NA	NA	NA
Sporer KA, et al. [35]	NA	NA	84 %	36 %	84 %	35 %
Feldman MJ, et al. [6]	34 %	32 %	68.2 %	66.2 %	80.3 %	50.7 %
Neely KW, et al. [36]	29 %	5.4 %	NA	NA	NA	NA

Scott et al. remind us that during the seventies, studies showed that the outcome of non-traumatic cardiac arrests was linked to rapid EMS dispatch [24, 25]. This observation paved the way to the ‘eight-minutes response’ that all dispatch centres within United States try to achieve for Advanced Life Support interventions. However, according to a 2002 systematic review [26], and to Snooks et al. when defining the highest priorities for research in emergency pre-hospital care in 2009, there is still insufficient evidence to generalize the effect of the prioritization of emergency ambulances on patient outcome other than cardiac arrest, and therefore it may not be a useful indicator [1, 27].

The future of dispatching may rely on a very structured interview based on questions with high PPV to avoid under triage, or NPV to avoid over triage for life-threatening conditions, combined with sophisticated computer algorithms to estimate better the patient’s life threat risk [28]. In a study from Japan, this system resulted in an over triage’s rate of 35 % and an under triage of less than 1 % for cases when looking specifically at cardio-pulmonary arrest cases (sensitivity 80.2 % (95 % CI: 78.6–81.8 %), specificity 96.0 % (95 % CI: 95.8–96.1 %), PPV 42.6 % (95 % CI: 41.1–44.0 %), and NPV 99.2 % (95 % CI: 99.2–99.3 %) respectively) [29].

### Over triage (Table 3)

‘Undefined problem’, which represents 38 % of over triage, can be used when the dispatcher did not get enough information from the caller, either because he/she did not ask the necessary questions, or because the caller was not near the patient or was too agitated to provide meaningful answers. It may also be used when a serious condition is suspected and dispatchers tend to quickly dispatch without paying too much attention to the categorization of the call. This keyword was replaced in our dispatch in 2012 by ‘disease not classified elsewhere’ and ‘trauma not classified elsewhere’ to slightly improve the description of the case, and therefore the quality of our case mix.

‘Public place’ represents 23 % of over triage. Historically P1 dispatch was required by the police department on those cases for security reasons. Since 2012, however, this has been suppressed to respect the classical dispatch criteria (mechanism, interview, clinical signs).

“Disturbance of consciousness’ represents 11 % of over triage. This keyword is associated with over triage 77 % of the time. Regarding the large spectra of non-specific problems that this term encompasses (orthostatism, alcohol or drug abuse, arrhythmia), its performance may be difficult to improve. ‘Dyspnoea’ and ‘chest pain’ represent 7 % and 8 % of over triage respectively. Finally, there were 247 cases for which P1 was requested by an on-call physician on site but that were classified with NACA score ≤3. Dispatchers are therefore not responsible for those cases of over triage.

We did not evaluate over triage between P2 and P3 priorities, as there is no official clinical criterion to discriminate these two dispatch levels. This choice mainly relies on the dispatcher’s clinical judgement. Furthermore we have not been able to set a standard to discriminate those priority levels as we did to discriminate P1 vs P2 and P3.

Seventy-eight per cent of over triage is not an acceptable rate and this needs to be improved.

### Under triage (Table 4)

With 446 missions, NACA 4 missions with the keywords ‘undefined problem’ (83.6 %) and dyspnoea’ (9.6 %) were the major sources of under triage. There are 36 cases (NACA >3) with on-call physician specifically not asking for a P1, most of them related to palliative care, which should not be considered as dispatch under triage. There are 8 NACA 7 cases that did not receive a P1 dispatch. This is due to voluntarily under triage by dispatchers when they believe death is certain or when palliative care is appropriate regarding information transmitted from the caller (spouse, nurse, doctor).

Although it has not been specifically measured in our work, we suggest that palliative care cases should be excluded from those studies if, at the time of the call, clinical instability is present but the level of priority dispatch chosen is low because of the patient’s choice or the decision by the physician on site.

It has not been demonstrated that under triage has any impact on patients’ outcome. “An accepted hierarchy of time-dependent interventions and thresholds for under triage is necessary for the judicious analysis and optimal design of a tiered EMS system” [5].

The ideal definition of under triage would be when an inappropriate priority level answer had an impact on the patient’s outcome. But this often remains subjective and therefore difficult to measure.

A first step allowing benchmarking between different EMS regarding over and under triage, would be to propose a list of clinical findings deserving dispatch with L&S. As all dispatch use L&S for high acuity case, this may allow the correlation of the perceived acuity by the dispatch centre with the clinical’s acuity on site, whatever dispatch system is used and whatever level of competences is available on board ambulances.

### Limitations

This is an observational retrospective study in a specific setting, not applicable to other dispatch systems or two-tier EMS.

We used the NACA score to evaluate the patient’s severity status, as this score has been significantly correlated with short-term survival, transfer to the intensive care unit and hospital length of stay [17–19] although it has been described as partly subjective [30] and not always reproducible [31]. The NACA score defines the most serious clinical state experienced at any given time during the mission. Therefore it may not always describe the patient’s clinical state on EMS arrival, as a minority of patients worsened during EMS care or transport, which may overestimate undertriage. A clinical evaluation on EMS arrival would be the best tool to evaluate dispatch’s decision.

All studies comparing a patient’s clinical condition at two different times (at time of dispatch and EMS clinical evaluation in this study) are subject to a limitation, as the patient’s situation can change for better or worse while EMS is on its way. Therefore, differences between the two time points may reflect an incorrect assessment by the dispatcher, or a change in the patient’s condition.

‘Undefined problem’ is the most used keyword in our dispatch in 2011. The overuse of this code (60 % of all missions), a much higher rate than in previous studies, lowers the quality of our case-mix description and illustrates the difficulties in describing and assessing emergency calls [8, 32].

## Conclusion

The rates of over triage and under triage in our CBD are 78 and 4.6 % respectively. The common estimation of high-acuity cases in the literature is 10 % of all calls, 14 % in our work. All centres should tend to achieve that proportion of high-acuity dispatches with the highest sensitivity and specificity possible, keeping in mind triage sensitivity and specificity are inversely related. The lack of consistent or universal metrics for the conduct of studies is perhaps the most important limitation in dispatch accuracy research. This is mainly due to the large heterogeneity of dispatch systems and competences of ambulance crews. A consensus on cases deserving dispatch with L&S could be a first step allowing benchmarking regarding over and under triage by dispatch centres.

### Ethics committee

This study was authorized by the Lausanne University Ethics Committee for human research.

## Abbreviations

CBD: Criteria based dispatch
ED: Emergency Department
EMS: Emergency medical services
L&S: Lights and siren
MPDS: Medical priority dispatch system
NACA: National advisory committee for aeronautics
NPV: Negative predictive value
PPV: Positive predictive value
